# Schoenlein's Clinical Lectures in the Infirmary at Berlin

**Published:** 1845-10

**Authors:** 


Schoenlein's Klinische Vortrage in dem Charité-Krankenhause zu Berlin: Redigirt und herausgegeben von Dr. *L. Guterbock.* Zweite Auflage. 8vo. pp. 480. Berlin, 1842-4.

Schoenlein's Clinical Lectures in the Infirmary at Berlin: Edited and published by Dr. Guterbock. 2nd Edition.

The name of Schoenlein stands high in Germany, and indeed throughout Europe, as that of a most enlightened and accomplished physician. The character and genius of these Reports will fully sustain the reputation which he enjoys; for few, we think, can rise from their perusal without gaining some instruction. They have reminded us in several particulars of those that were published, some years ago, by Dr. Bright, and which at once established the fame of that gentleman as one of the shrewdest observers and ablest practitioners of the day. German works are usually so prolix and wearisome, and withal often so intricate and perplexed, from the spirit of abstruse disquisition which pervades them, that it is really quite a treat to meet with such a production as the present from our trans-Rhenal brethren. Hitherto, it is truly astonishing how little has been contributed (within the present century) to the advancement of *practical* medicine—we do not say, surgery—by a people whose men of literature and science are universally looked up to as patterns of Herculean industry and unwearied research. But it may be the very possession of these most meritorious attributes, that has in some degree disqualified them from being simple observers and practical therapeutists. They are so much taken up with searching into the *causes* of disease, that they often entirely neglect the immediate *effects* (which are the symptoms); and, in their zeal to pry into the hidden and profound mysteries of vital action, they are not unfrequently apt to overlook what is lying before their very eyes upon the surface. It is the fashion of some writers to decry the unphilosophical (as they choose to call it) method of seeking to cure diseases by treating symptoms, without having constant regard to the proximate causes from which they may be supposed to spring. This might be all very well, if diseases were uniformly the same entities in all individuals, following precisely the same course and always amenable to the same remedies. But such is not, and cannot from the very constitution of our minds and bodies, be the case; and, whatever theorists may choose to say to the contrary, that man will always be considered to be the most skilful physician who knows best how to afford relief to present distress, and, at the same time, "to obviate the tendency to death," in all grave maladies. Let it not be supposed that we under-rate the value of the aids to be derived from the pursuit of morbid anatomy, of the microscopic and chemical examination of healthy and diseased structures, and of whatsoever is calculated to make us better acquainted with the intimate organisation of animal bodies; all that we mean to contend for is, that no undue or exaggerated importance should be attached to such researches, as if they were unerring guides to the practitioner in the treatment of disease. They may, indeed, *suggest* the use of remedies, but they must not be allowed to *dictate* them. They may often teach us how to *navigate* the vessel, but not how to *steer* it; especially when it is tossed about by baffling winds, or when it is approaching a dangerous and uncertain shore. The best pilot will be he who knows not only the bearings of the land, the depth of the soundings, the drift of the currents, and so forth, but also how to work the ship most efficiently, to guide the helm, to trim the sails, and, in short, to make use of all those dexterities of seamanship, which nothing but experience and patient assiduity can give. Now, it is in the dexterities of medical practice that the English physician is generally believed—and, we think, with perfect justice—to be superior to his continental brethren; he is not so learned a navigator, but he is a better steersman of the vessel of life through many of the difficulties of its stormy and perplexed voyage.* The author, whose opinions we are now about to bring under the notice of our readers, is a man of this practical stamp; and, as he has been long engaged in the active duties of the profession, it cannot but be useful to compare notes with him, keeping our minds free from all partiality or prejudice, not tied or *addicted* to the precepts of any one school or master; in short, willing to receive, and ready to communicate. We may, therefore, cordially reciprocate the Horatian sentiment:

> Vive, vale. Si quid novisti rectius istis,
> Candidus imperti; si non, his utere mecum.

---

* It is always amusing, and may be instructive, to learn what strangers say of us as a people. The following is one of the latest descriptions of our character and manners by a foreign traveller! The English are "a people who can only contrive to get rid of their *ennui* by plunging into the vortex of political and mercantile activity. The perfection of the machines, which are here placed everywhere in bustling operation, and perform so many of the functions of human beings, made a disagreeable impression upon me. This artificial bustle of wheels, bars, cylinders, pistons, thousands of little blocks, pegs, teeth, &c., which move almost with passion, filled me with loathing. The determinate, the exact, the measured, and the precise, in the life of the English people, tormented me no less; the machines appear to me like men, and the men appear like machines. Wood, iron, and brass seem to have usurped in England the functions of living man, and to have absolutely become insane from a superabundance of the spiritual principle; whereas, the spiritless man, like an empty and inanimate spectre, performs his usual routine of occupations after the manner of a machine; and at the exact predetermined minute, he eats his beef-steak, makes a speech in Parliament, steps into a stage-coach or railway-train, goes to bed, or hangs himself." Poor John Bull!—foreigners don't like you, that's clear.

The entire volume is taken up with reports, more or less detailed, of medical cases (42 in number); the diseases, that are illustrated, being Typhus Fever, Pneumonia and Pleuritis, Rheumatism and Heart-affections, Peritonitis, Jaundice, Ileus, Hæmatemesis, Tumours in the Abdomen, Diabetes, Erysipelas, Apoplexy, &c. As the subject of Typhus Fever is treated at greater length than any other disease, we propose to give abstracts of most of the cases that are related, selecting such expository remarks and comments as appear to be most useful and instructive.

*Typhus Abdominalis—Injurious Effects of Emetics—Utility of Calomel—Deterioration of the Symptoms after the* 14*th day—Coma and Death—Dissection—Remarks.*

A youth, 19 years of age, exhibited upon his admission into the hospital the usual symptoms of *Typhus mitior*. The abdominal symptoms were not very strongly marked; for there was scarcely any tenderness on pressure, even over the *caput cæci*. The urine was turbid, and had a mucous sediment. The patient had been seized with shiverings, eight or nine days before; so that he might be considered as in the second week of the fever.

It used to be the practice of many of the older physicians to administer an emetic at the commencement of Typhus fever, with the view of cutting it short. Dr. Schoenlein condemns this practice; according to his observation, the gastric and other symptoms have generally become aggravated after the action of vomiting, more especially when the tartrate of Antimony had been given. As the eruption of an Exanthematous fever is always found to be the greatest upon any part of the surface of the skin which may have been accidentally irritated, so we may reasonably infer that the eruption or exanthem upon the mucous surface of the alimentary canal must be aggravated by the irritation which an emetic necessarily produces. For the same reason, saline purgatives are generally injurious in the treatment of Typhus: Calomel is a much safer remedy. Autenrieth, in 1806 and 1807, first recommended its exhibition in this disease: he gave it in small doses, for the purpose of causing the peculiar green stools that usually follow its administration. Formerly, alvine evacuations of this character were supposed to contain a quantity of Bile; but recent microscopic researches seem to shew that it is vitiated Blood, rather than bile, that is present in them, giving them their peculiar appearance. During the administration of Calomel, the motions often become more solid, and less frequent, than they were before; so that we may even have occasion to give enemata in order to procure sufficient evacuations—a sufficient proof of the unirritating properties of the medicine. Very different, we need scarcely say, is the action of saline purgatives.

In what period of Typhus is the use of calomel most beneficial? Dr. Schoenlein would limit its use to the first eight or nine days of the fever; and he remarks that, the earlier it is given, the more useful are its effects: the best period for its exhibition being the first three or four days. The accession of great tenderness of the abdomen, with a dry skin and a frequent pulse, and the aggravation of the nervous symptoms, indicate the limit to its further exhibition. In a later period, its administration is positively injurious. Physicians differ a good deal as to the doses that ought to be given. Some order three or four grains every two or three hours, until the desired alvine evacuations are induced; while those of the Tubingen School prefer to exhibit a full scruple of the medicine at once, repeating the dose every second day afterwards, until the motions become less frequent. In the present case, the latter plan was followed. The bowels were not much moved in this patient by the large doses administered. As there is often an acid present in the stomach, it will be generally found useful to combine the Calomel with a few grains of Magnesia. Dr. Schoenlein considers that salivation, so far from being desirable in Typhus fever, is always to be regretted. If the Calomel is not brought in immediate contact with the mouth, when it is swallowed, there will be less tendency to this unpleasant effect taking place. A weak solution of Iodine is one of the most useful correctives of mercurial salivation.

The second dose of the Calomel (℈j.) caused four evacuations, which were of a brownish and dark green colour, and of a pappy consistence. As the Skin is the organ, by which the critical resolution of Fever usually takes place, small doses of *spiritus Mindereri* were ordered to be taken frequently, and a warm bath also was prescribed.

About the end of the second week, and therefore as one of the critical days drew near, there was observed to be a greater degree of stupor than had hitherto existed: the tongue was furred, but moist; the pulse was moderate; and the abdomen continued soft, although in the cæcal region there was always the characteristic gurgling murmur, and also a slight tenderness in the part upon pressure. The warm-bath was repeated; the diaphoretic medicine, in rather larger doses and with the addition of a little *tinct.* Valerianæ, was continued; a mustard poultice to the calves of the legs was ordered; and also an aperient enema, as the bowels had not acted for 48 hours.

So far from any decided resolution taking place on the 14th day, the nervous symptoms had become rather worse; and there was therefore now no prospect of any decided amendment taking place for another week. The patient was in that state which P. Frank characterizes as "nervosa stupida:" he was more or less oppressed during the whole of the day, passing his water in bed if not roused; and he was somewhat delirious during the night. A cold lotion was applied to the head; some leeches were put upon the temples; an enema with the *acet. plumbi* was administered;* and the *infus. Valerianæ* with *Mindererus spirit* given in frequent doses. Matters did not mend; the stupor increased; and there were now frequent twitchings of the muscles, and grindings of the teeth. The abdomen was more tender upon pressure; the motions were more feculent, but not more frequent, than before. As the disease had now assumed an altogether torpid character, the *infusum Cinchonæ* was given, and blisters were applied upon the calves of the legs. In spite of these means, the

* This enema consists of gruel, to which 10—12 drops of the *liquor plumbi* are added: if there be any diarrhœa present, the addition of a small portion of Laudanum will be useful. The longer the patient can retain the injection, the better. It is a favourite prescription of Schoenlein's.

unfavourable symptoms increased, the patient became more and more comatose, and died on the 17th or 18th day of the disease.

*Dissection.*—A number of small scrofulous glands or tubercles were found in the *omentum*, and peritoneal covering of the small intestines. On the mucous surface of the lower end of the *ileum*, and of the commencement of the *colon*, there were several small ulcerated points, which seemed to have been in the act of healing at the period of death. The Peyerian glands were but little affected. The intestinal lesions in this case were therefore much less decided than is usual in Typhus fever. The lateral ventricles of the *brain* were distended with serosity; and there was also a yellow-coloured gelatinous deposit under the *pia mater* at the base of the brain.

Dr. Schoenlein attributes the death in this instance to the cerebral changes. He considers that the appearances found in the bowels—viz. the traces of healing in the ulcerated points on the mucous surface—may fairly be considered as evidences in favour of the use of Calomel: although it does not *prevent*, it seems to *moderate*, the severity of the intestinal eruption.

(*Remarks.*—There are several points in this case that deserve notice. Schoenlein's condemnation of emetics in the early stage of Typhus, and his reasons for so doing, are not at all satisfactory. In our opinion, they are quite fallacious, and appear to be founded much more on his theoretical views of the disease, than upon the results of actual observation. Nature herself often points out the practice that should be followed; and how decided is the relief that is generally obtained by free and copious vomiting! For our own parts, we cannot too strongly recommend the administration of an emetic at the commencement of almost all fevers. The practice of giving scruple-doses of calomel in mild cases of Typhus is not likely to find favour with the British physician: moderate and repeated doses of the hydrarg. c. creta, to which a few grains of carbonate of soda may be added, with grateful effervescing draughts in the intervals, will be generally preferred.—Schoenlein seems to be haunted every moment with the danger of *dothinenterite;* and yet his patient died, he admits, of cerebral effusion, and not of any intestinal lesion.—Query. Ought the case therefore to be called one of *typhus abdominalis?*—Blood-letting, in any form, is seldom or ever admissible for the relief of the comatose affection in the advanced stage of Typhus.—The doctrine of "critical days," is, it will be observed, much insisted upon.)

Second Case.——*Typhus Abdominalis—Thoracic Affection—Epistaxis—Emaciation of Typhus Patients—the Blood in Typhus and in Chlorosis—recovery.*

A young man, when admitted, exhibited the usual symptoms of mild Typhus. The cerebral disturbance having been at the first more severe than the abdominal, he had been bled from the arm in consequence. There was little or no pain, but only a rumbling, in the cœcal region. A slight pneumonic affection existed on the left side: it was indicated by a somewhat increased dulness upon percussion, by the presence of a moist rhonchus, and by the sputa being a little tinged with blood. As the fever had already existed nearly 14 days, the treatment was directed chiefly with the view of promoting a critical eczumation from the Skin by the use of mild diaphoretics; and, for the purpose of relieving the pulmonic disorder, 2 grains of *Muriate* of *Ammonia,* with $\frac{1}{8}$ of a grain of *Camphor,* were given every two hours. Two days subsequently, an Epistaxis supervened: Schoenlein regarded it as of a "critical" nature. When this hæmorrhage is excessive, he advises that the nostrils should be plugged with lint dipped in *Aqua Thedeni,* that Alum be administered internally, and that sinapisms be applied to the surface to produce irritation of the peripheral nervous system. In the present case, these remedies were not called for. For the purpose of acting upon the skin and lungs, the *mur. ammon.* with *sulphur. aurat.* was given; and some mercurial ointment was rubbed in over the lower lobe of the left lung. But, as next day the chest symptoms were rather increased than diminished, a few ounces of blood were then drawn from the affected side by cupping: the blood thus obtained was very thin and watery: when examined with the microscope, it was found to contain a much smaller proportion of red globules than exists in healthy blood.* The *objective* (auscultatory) as well as the *subjective* (rational) symptoms were decidedly relieved by this practice. In reference to the very mild character of the abdominal symptoms in the present case, Schoenlein remarks:

"The alvine evacuations are in no way pathognomonic in this disease, as some writers have maintained; and it requires that we should be well acquainted with the anomalies that are apt to occur, in order that we may not, by their mere absence, be led into error. Notwithstanding the long (for three days) constipation, and the absence of pain or any rumbling noise in the region of the cæcum, there are doubtless (in the present case) certain lesions of the intestinal mucous surface present."

---

* Schoenlein on this occasion remarks that the result of this examination was quite in accordance with his opinion as to the nature of Typhus fever, which he regards as "a blood-disease or a hæmatosis, essentially distinct from inflammation." Besides a deficiency of Fibrine and Albumen, the proportion of the red globules is often very sensibly diminished in Typhus. The blood in the present case was examined by Dr. Simon, and the following are the results of his analysis.

| Healthy Blood. | | Blood of this Patient. |
|---|---|---|
| 791 | Water | 887,5 |
| 208 | Solid contents | 112 |
| 2,0 | Fibrine | 0 |
| 76 | Albumen | 54 |
| 112 | Blood-globules | 47,25 |

There are several reasons that make us believe that there is a decided change in the colouring matter of the blood in Typhus—viz: the formation of the dark pigment on the teeth, lips and tongue, the stained appearance of the inner surface of the blood-vessels and of the intestines, and especially the state of the urine. Dr. Simon has found, in the deep-coloured urine of a Typhus patient, the colouring matter of the blood and uric acid in combination with a basis.—Dr. Schoenlein accounts for the greater emaciation and the much slower recovery of the strength after an attack of Fever than of Inflammation, in which a large quantity of blood may have been lost, by the circumstance of the greater *deterioration* of the blood in the former instance.

It here deserves to be remarked that the physician should never trust to the mere feelings of a patient, or to the answers which he gives to questions, touching the existence and severity of his abdominal and thoracic symptoms; for these are often, as it were, disguised, and thus are apt to be sometimes entirely overlooked. Nothing, therefore, but an "objective examination," every day, should satisfy the judicious practitioner. This is more especially necessary, as the visceral affection is often observed to oscillate, or alternately to subside and to be excited, several times in the course of an attack of Typhus fever. In the present case, after a *lull* of five days, the thoracic affection was again aggravated, the *rhonchus sibilans* being again heard on the left side, and being now accompanied with a slight crepitating noise. As it would seem from this that the Bronchitis was in some degree complicated with Pneumonia, 8 ozs. of blood were drawn from the arm, the cupping-glasses applied to the chest, and frequent doses of *Nitre* and *Sal Ammoniac* administered. Under such circumstances, we must be on our guard lest the pneumonic inflammation pass into the suppurative process; for this is an accident that is occasionally observed to occur in protracted typhoid affections. The symptoms were considerably relieved by the use of the remedies employed; the *sibilant* had changed more into the *mucous rale*, &c. But, as the cough was still dry, harsh and ringing, and as no decided critical evacuation, nor any local crisis had taken place, it was deemed advisable to have the mercurial ointment rubbed in upon the chest, especially between the shoulders. What we desire to find is, that the moist rhonchus may be heard over a wider extent of the chest, also that the cough may become looser, and that the sputa assume a more globular and distinct character. The febrifuge expectorant mixture—half-a-drachm of *nitre* and the same quantity of *Sal ammoniac* in six ounces of emulsion—was ordered to be continued. The symptoms gradually gave way, and the patient eventually was restored to health.

Schoenlein remarks that it has been only of late years, that the attention of the physician has been specially directed to the not unfrequent existence of a latent pulmonic affection in cases of Typhus fever. The *subjective* symptoms of this complication are usually not very prominent or striking; and it is therefore only by the aid of the *objective* phenomena, as disclosed by Auscultation, that our diagnosis can be accurately formed. It is to M. Louis, more than to any other pathologist, that we are indebted for our more exact information upon this subject. He has most convincingly shewn how apt Pneumonia is to supervene, when we little suspect it. Too often it passes on into phthisical disease, especially in those persons in whom there exists any constitutional tendency to this malady. The frequency of chest complications in Typhus varies much in different seasons. Hence the importance of studying the epidemic and endemic constitution of each fever, with which we have to do.

(*Remarks.*—In spite of his bias to the abdominal pathology of Typhus fever, Schoenlein expressly recognises the importance of studying the changes and abnormnal condition of the fluids.—The occasional complication of pulmonic inflammation, it is of the highest importance to be aware of. In the treatment of the present case, we miss the use of two very important remedies—the tartrate of Antimony, and Blisters).

In the *third case* of (what is still called) " abdominal typhus," the points most worthy of notice are these. An emetic had been given to the patient before his admission into the hospital; and, as after its administration a loose state of the bowels supervened, Dr. Schoenlein inferred that it had accelerated the eruption upon the intestinal mucous surface. Had this not been the case, he remarks, we might have hoped to have prevented its development by the use of Calomel: the disease having reached that point when its resolution by some critical evacuation is still possible, but cannot be, as it were, compelled. *Aqua oxymuriatica,* in a demulcent beverage, was ordered to be taken frequently.

There was an unusual tendency to exhaustion and syncope in this patient whenever he sat up. This peculiarity Schoenlein regarded " as a pathological equivalent of the intermittent fevers, which are strikingly rare this Spring." " I have," he continues, " at the present moment under my care a patient, in whom his usual Spring attack of Ague is replaced by a regular and periodic return of faintishness."*

*Relation between Typhus and Intermittent Fevers.*

That Typhus Fever and Ague are often very closely associated, more especially as respects their genetic cause—the former so frequently springing from the latter—is well known to those who have practised in marshy districts. The truth of this remark was abundantly witnessed in the great epidemics, which prevailed in the North of Europe during the years 1827 and 1828. One example will suffice. Where the Rhine opens into the Bodensee, the stream of the river becomes as a matter of course nearly still, and there Malaria is generated and Agues abound ; but in Appenzel, where the land becomes more elevated, there are no Agues, but often a most destructive form of Typhus fever prevails.

This subject of pathological relationship is one of such high practical importance, and withal so little attended to, that we shall make no apology for introducing the following remarks by our enlightened author.

" These equivalents of intermittent fevers, as we may call them, generally shew themselves in this manner. The affection appears at first only as an inconsiderable irritation of the gastric or bronchial mucous membrane, perhaps as a trifling catarrh; but there is always—and this symptom may have existed for some days previously—an unusual degree of powerlessness and general oppression. In young plethoric subjects, there is not unfrequently at the same time a discharge of blood from some part, to a greater or less extent: in women, it is often an excessive flow of the menses—all indicative of a vitiated state of the fluids. In such circumstances, let the physician be on his guard: for it is not with a simple catarrh that he has to do. If, having regard only to the loaded tongue, nausea, and headache, he administers an emetic or saline purgative, the

---

* This truly Sydenhamian remark well deserves attention; and we are the more anxious that our readers should notice it, from its being so completely in accordance with those highly instructive observations of Dr. Harden, on what he has called the " Isopathia or Parallelism of Diseases:" vide our last No. p. 277. The very expressions used by the two writers are *equivalent*.

chances are that he inevitably hastens on and aggravates the development of the disease. During various epidemics, many cases have been observed by me where the patients have complained of little save weakness and tendency to fainting, without perhaps any symptoms of intestinal disturbance or any febrile excitement. Such cases were well known to the old physicians, who have described them under the appellation of *febres epidemicæ malignæ sine febre.** Not unfrequently about one of the critical periods, usually the end of the third week, a violent vascular tumult (gefass-sturm) is excited; and then either quickly subsides by some critical evacuation from the skin, kidneys, or bowels; or a rapid and perhaps fatal collapse ensues."

(The modifying influence of an agueish character or constitution upon the progress of continued Fevers is, as might be expected, most conspicuous in hot climates. As the modern French school of medicine is that which has most grievously erred in overlooking this important point of practice, we are always pleased to meet with evidences of sounder therapeutic views in the writings of our neighbours. The following passage, from a recent work on the Diseases of Brazil, may be not inaptly introduced as a specimen of the wholesome change that is beginning to take place in their pathology.†

" Experience has taught me, as well as others, to modify my opinions, and change my therapeutics, in the presence of a host of grave fevers, which I used to regard as symptomatic of inflammatory lesions of the liver, brain, or intestines. It has convinced me that, in those countries, where the *agueish principle* predominates, every thing is subjected to its action; and therefore that to persist in the employment of antiphlogistic remedies in those countries, especially when an epidemic of grave intermittent fever exists, is to cause almost inevitably the death of our patients. For the last twelve years, I have been satisfied of the truth of this: before that time, I held the exclusive doctrines of Broussais, who used to quote me as a zealous follower of his doctrines in South America. It has cost me much regret to separate myself from the school of this puissant genius, but facts speak louder than any doctrine can do; and I am bound to proclaim the infallible efficacy of the sulphate of Quinine in the treatment of intermittent fevers, even in their acute stage.")

Case 4.—*Typhus Abdominalis, nervosa, stupida—Pneumonia—Epistaxis —Threatening of Œdema Glottidis—Ammoniacal Urine—Parotitis— Recovery.*

A man, 23 years of age, was admitted into the hospital, with the ordinary symptoms of Typhus: according to his report, he was in the beginning of the second week of the fever. The headache had been so severe as to require the application of leeches. Although the patient made no complaint of any thoracic ailment, Percussion and Auscultation shewed that there was incipient inflammation of the posterior part of the right lung; the sound that was elicited over it being duller than usual, and a

---

* It is in such cases, where the symptoms are so obscure and ill-marked during life, that *inflammationes occultæ* in the viscera are so often met with upon dissection.

† Du Climat et des Maladies de Bresil, ou Statistique Medicale de cet Empire, par J. F. Sigaud, Medecin de S. M. l'Empereur dom Pedro II. Paris, 1844.

distinct crepitation being audible whenever a deep inspiration was taken. As usual with cases in which the cephalic symptoms are dominant, the abdominal ones were indistinctly marked. Schoenlein dwells, very emphatically, upon the importance of the medical attendant not allowing his judgment to be influenced solely by the amount of uneasiness or the degree of pain in the abdomen ; as he has repeatedly seen, upon dissection, most serious and extensive intestinal lesions in cases where the abdominal symptoms had been masked or obscured by the predominance of the cerebral oppression. It is this form of Typhus, of which the present case is an example, that Frank characterises as the *febris nervosa stupida.* In consequence of the thoracic symptoms, the patient was ordered to be bled to ten ounces from the arm, to be cupped over the inferior part of the right lung behind, and to take repeated doses of nitre in an oily emulsion.

On the subject of *blood-letting in Typhus fever,* Dr. Schoenlein thus expresses himself.

"I do not partake the opinion of those who would make this fever to consist in an inflammation of any one part or organ of the body, whether this be supposed to be the brain, or the entire nervous system; or the mucous membrane or glands of the intestines; or the endocardium, &c.; nor can I admit, with some writers, that it is to be cured by the use of blood-letting. The doctrine of Bouillaud is downright nonsense, and his practice is little better than murder. And yet it is no doubt quite true that the *genius* of some epidemics is decidedly inflammatory, and may require tolerably copious depletions.

"There are cases in which blood-letting cannot be dispensed with, in consequence of an existing inflammatory affection of certain internal organs. But the greatest judgment is required in its adoption. I have seen cases in which, in consequence of the fever setting in at first with all the symptoms of Encephalitis, free blood-letting was practised; and the result was a rapid collapse, followed speedily by death. In one case of this sort, not the slightest trace of cerebral mischief was discoverable on dissection, and the only lesion found was the usual typhous affection of the bowels. In exanthematous fevers, and especially in Small-pox and Scarlatina, the head-symptoms are often very severe before the eruption has made its appearance on the surface. Perhaps something similar takes place in Typhus. In all cases, therefore, where the headache is very severe, it is better, first of all, to cause a derivation of the vascular action by means of sinapisms to the feet, and of enemata of vinegar, than to have immediate recourse to sanguineous depletions. The only circumstances, which can justify the employment of venesection in Typhus, are the accession of Pneumonia, or the existence of strong pressure upon the Brain."

And even when these phenomena are present, it will much depend upon the general character of the existing epidemic, and on the constitution of each individual patient, whether we should have recourse to general or local blood-letting to relieve the oppressed viscera.

In the present case an Epistaxis supervened, and all the cerebral symptoms were observed to be subsequently much relieved : this hæmorrhage, therefore, was of a truly critical nature. There being still some degree of mucous *rale* perceptible over the basis of the right lung, small doses of *Sulphur. aurat.*, in combination with *sal ammoniac* were ordered. In proportion as the thoracic and cephalic symptoms subsided, those appertaining to the abdomen became more conspicuous : the tenderness of the cœcal region, its gurgling upon pressure, and the setting in of a slight diarrhœa, abundantly testified where the chief peccant cause now lay. The *alum-whey* (which had been prescribed for the Epistaxis) was continued; clysters of starch with opium were administered; and mercurial ointment was rubbed in over the region of the cæcum.

*Necessity of a guarded Prognosis in Fever.*

As long as the Typhoid process exists, the physician should be on his guard not to regard his patient as convalescent, however mitigated the existing symptoms may be. A complication—as, for example, an inflammatory condition of the lungs—may be entirely absent or subdued to-day; and, in the course of two or three days, it may return without any appreciable exciting cause. Thus it was in the present case. After some days entire subsidence, the auscultatory pneumonic symptoms returned; but now upon the *left,* not the right, side. Some blood was taken from the part by leeches, with marked advantage. The importance of the Stethoscope, in regulating our treatment in many cases of Typhus, cannot be too highly estimated.

A slight aphthous state of the fauces having made its appearance, a gargle, composed of Chlorine solution and Alum, was ordered to be used frequently. Next day, the left tonsil was observed to be swollen and œdematous. The progress of this guttural affection should always be very narrowly watched, as one of the most formidable diseases, to which mankind is subject—*œdema glottidis*—may, under such circumstances, suddenly supervene, when no danger is apprehended. It is especially apt to occur in certain exanthematous fevers; viz. Small-pox, Scarlatina, and Measles. Dr. Schoenlein regards the use of acid and alum gargles as one of the most efficacious means, if early employed, to arrest the progress of this most dangerous affection. We may here remark that it is by no means uncommon for an aphthous or ulcerated condition of one or more of the mucous outlets—the nostrils, throat, eyes, ears, rectum, and vagina—to occur during the progress of, and convalescence from, an attack of fever.

As there was a threatened relapse of the Typhoid symptoms—uneasiness and distension in the cœcal region, dry skin, accelerated pulse—the *aqua oxymuriatica* was prescribed, and a tepid affusion was ordered to be used at bed-time. The patient not being relieved next day, 12 leeches were applied over the cæcum, and the warm-bath repeated. The great frequency of the pulse at this time rendered the prognosis more unfavourable than we might have been inclined to come to, judging from the other symptoms; for it may be laid down as a general position, that, whenever the pulse keeps above 120, we have much cause to apprehend the ultimate result of any fever. Nevertheless there are exceptions to the truth of this remark; and it is therefore only by carefully attending to all the symptoms collectively, and not to any one in particular, that the physician can form a correct judgment of the probable issue.

No sooner had the guttural and abdominal affections subsided than symptoms of Bronchitis, on the right side, made their appearance—dry sibilant rhonchus, and distressing cough: the sputa were not at all tinged with blood. The cupping-glasses were applied over the affected part, and small doses of *nitrate of soda* and *muriate of ammonia* were given every two hours. Next day, the left parotid gland (it is curious that Parotitis and also suppuration of the internal Ear are much more frequent on the left, than on the right, side) was swollen and inflamed. Schoenlein recommends the application of leeches and mercurial frictions for the relief of this symptom: he disapproves of rubefaciants and other local irritants.

The Urine, which in the early stage of this case was deep-coloured and strongly acid, had now become sedimentary and so alkaline that, when a glass-rod dipped in muriatic acid was held over it, vapours of muriate of Ammonia were immediately perceptible. This state of the urine was well known to the old writers, who termed it "lixivial." Whether it is connected, in such cases as the present, with an injury of the functions of the cerebro-spinal axis, we are not prepared to say; certain it is that it is a not uncommon attendant upon lesions of the great nervous centres. The deposit was found, by the aid of the microscope, to contain numerous crystals of urate of ammonia, and of the ammoniaco-magnesian phosphate. The *aqua oxymuriatica* and a mild *decoction of cinchona* were prescribed; and light nourishing food was given frequently. In all cases of protracted Typhus, where the strength and flesh are much wasted, it will be prudent that nourishing clysters of milk, soup, &c. be regularly administered. As we have remarked before, the loss of power and substance, which the system sustains during the progress of a continued fever, and the consequent emaciation and exhaustion that take place, are much more considerable than after almost any other disease. Before the convalescence of our patient was fairly established, the right parotid gland also became inflamed and threatened to advance on to suppuration. For a considerable time the pulse remained at a high rate, and there was always more or less cough each night and morning; these symptoms demand, as a matter of course, watchful care; as there is very often a decided tendency to the setting in of phthisical disease after Typhus, as well as after the Exanthemata. In the present case, they gradually subsided, and the patient was eventually restored to perfect health.

*The varying Conditions of the Urine at different times of the same day.*

In reference to this point—which is one that demands the assiduous attention of the physician—the following remarks may be read with profit.

"The urine voided in the evening exhibited very different characters, chemical as well as physical, from that which was passed in the morning; the former was turbid and alkaline, while the latter had an acid re-action, and was of a normal appearance. Unless attention be paid to the state of the secretions at different periods in the twenty-four hours, the practitioner may be led into very serious errors. In affections of the Liver and Spleen, we have often occasion to observe the important fact, that the urine, voided after a meal, exhibits abnormal characters, while at other times it may seem to be altogether healthy. In affections of the Kidney too, we also find—which we should certainly not expect—this peculiar appearance: the urine, which is voided at night, frequently contains a considerable quantity of matter: while that, voided during the day, may contain little or perhaps no trace of it. In Diabetes, the urine passed at bed-time, and after a meal, often contains much saccharine matter; and yet there may be scarcely any trace of it at other times of the day. The old physicians were much more in the habit of attending to these varying phases of the urinary secretion than the modern ones. In the case at present under consideration, the urine passed at bed-time continued to exhibit the *critical* condition which had been noticed for several days previously; whereas, that passed in the morning, appeared to be altogether healthy."

The *fifth* case was one of Typhus *nervosa lenta* vel *versatilis*,* characterised chiefly by distressing irritability of the nervous system, without, however, the symptoms of any grave lesion of the brain, chest, or abdomen. Schoenlein recommended clysters composed of *infus. Valerianæ* and *Castoreum*, and the following mixture to be taken in ounce doses every two hours.

℞. Infusi Valerianæ (ʒij.) ℥iv.

Mucilag. Salep. ℥ij.

Acid. Muriatic. ʒj.

Syrupi Simpl. ℥j.

Opium is in most cases of this sort not only useless, but positively hurtful.

In recovery from this form of Typhus, our author remarks that there is not unfrequently an inordinate excitability of the generative organs; and he recommends, in consequence, that not only the patients, but also that their nurses, should be carefully watched. "I have so often," says he, "had occasion to witness the misfortune of this precaution being neglected, that I cannot insist upon it too much."—In some cases, during the period of convalescence, the craving for food is so great that it amounts to *bulimia*, and must be regarded as a positive disease. Under such circumstances the physician should very narrowly watch the progress of the case, especially if there is reason to believe that the intestinal ulcers are not completely cicatrised. On this subject—one of great practical as well as pathological importance—we are presented with the following observations by our experienced author.

### *The Cicatrisation of the Intestinal Ulcers.*

"The process of the healing of intestinal ulcerations, in cases of Fever, usually commences about the end of the third week, and continues for a greater or less length of time, in proportion to the severity of the typhoid disease. In mild cases, it is generally completed by the end of the fourth or fifth week; but in severe ones, not for three or four weeks afterwards. The patient cannot be considered out of danger, until this process be entirely over. Before this period, how easily are the typhoid symptoms renewed by any irregularity of diet, by exposure to cold, &c.!—not that the relapse is owing to the development of any new lesion of the intestinal surface, but only to the fresh irritation that has occurred in and around it, and which is accompanied with an asthenic fever. In some unfortunate cases, the

---

* Reil has described what he calls "Irritable Typhus;" the most prominent characters being great sleeplessness, loquacity, twitching of the tendons, &c.: the urine is generally clear and abundant. Occasionally, severe crampy affections of various muscles will occur in the course of such fevers. If the Heart or Larynx, or Diaphragm be so affected, death may take place most unexpectedly. Trismus, Tetanus, and even Hydrophobia, with its peculiar convulsive difficulty of swallowing, have been known to occur and prove rapidly fatal. Schoenlein alludes to a case where the muscles on the back of the neck were affected with most distressing involuntary movements: at one time it was apprehended that cerebral disorganisation was going on; but this alarm proved groundless.

ulceration eats deeper and deeper, until a perforation is made through the intestinal parietes.

* * * What are the phenomena which indicate that the process of intestinal cicatrisation is completed?—1, A cessation of all tenderness and rumbling noise in the right iliac region after eating, as well as at other times; 2, the disappearance of pus-globules from the alvine evacuations; 3, the cessation of all feverishness in the evening, and just before midnight; and 4, the arrest of further emaciation, and the gradual recovery of flesh and strength."

(These observations should be read, marked, learned, and inwardly digested by every medical man, who desires to carry his patients through the dangers of the convalescent, as well as of the earlier stages of typhus fever. Whether there be intestinal ulcerations or not—and we are decidedly of opinion that they are by no means so constant phenomena as Schoenlein believes—too much attention cannot be paid to ascertain the condition of the abdominal viscera, by daily examining the abdomen with the hand, and watching the state of the evacuations during the recovery from this insidious disease.)

CASE 6.—*Typhus Abdominalis—different Kinds of Cerebral Irritation in Fever—Affection af the Larynx—Episodes in the course of Typhus—unequal Distribution of Temperature—Death—Dissection.*

In the present case, the abdominal symptoms were the most prominent, and were evidently those which threatened the chief danger, although the lungs and brain were not entirely exempt from disturbance. A dozen of leeches were applied over the cæcum; and the *aqua oxymuriatica* administered in injections as well as by the mouth:* a warm-bath, after the leeches, was ordered. The stupor and cerebral oppression rapidly increased in gravity, and consequently attracted much of the attention of the clinical professor. The following observations on the *nervous or cerebral symptoms in Typhus fever* were suggested by the state of the patient at the time:—

" I am satisfied that they spring from very different causes, and therefore require very different treatment in different cases. 1. There is an irritation of the Brain and nervous system which usually appears on the third or fourth day of the disease, and which, in my opinion, is contemporaneous with the development of the intestinal eruption; it may therefore be regarded as altogether analogous to the cerebral disturbance that usually precedes and accompanies the appearance of the rash or pock in the genuine exanthemata. This affection of the brain is usually manifested by symptoms of acute excitement; such as delirium, redness of the face, hot skin and full pulse. It subsides or altogether ceases as soon as the eruption of the intestinal mucous surface has taken place; this is generally about the seventh day. 2. A second form of cerebral irritation occurs at a later period, and depends upon a state of congestion that, in its nature, approaches a good deal to that of Inflammation. When present, the fever exhibits the type of what has been called *cerebral Typhus*. It often appears early; but in some cases not till the seventeenth or eighteenth day. 3. Another kind

* This acid was given, it is stated in the report, to counteract the general or constitutional morbid process:—as an antiseptic or corrigent of the vitiated fluids, we suppose.

evidently proceeds from the abdomen as its origin; depending on the formation of ulcers in the bowels, just in the same manner as cerebral irritation in children is so often connected with the presence of Worms. 4. A fourth form occurs at a later stage of the disease, when the crises have already taken place; this form is similar to the cerebral irritation in persons who have suffered from profuse discharges. The prominent symptoms are delirium and wakefulness.

"The *treatment* of the inflammatory or hyperæmic forms, especially of the *second* enumerated above, consists, as a matter of course, in the use of local blood-letting, cold lotions, and derivatives; of the *third*, or abdominal form, in the employment of sedatives and anti-spasmodics, given in injections as well as by the mouth; and of the *fourth* in the exhibition of bark, nourishing food, &c.

"It thus appears that the same phenomenon, occurring in the course of the same morbid process, may have a very different indication in the eye of the practical physician; and it is therefore a perfect folly to regard it always as of the same nature, in the manner that Marcus and so many of the modern French school have done. It is of paramount importance that, in each individual case, good care be taken to make out the real nature of the existing cerebral irritation, so as to determine upon the line of practice to be adopted. In the present case, it is obviously connected with the affection of the intestines; and accordingly, an enema of an infusion of *valerian* with *castor*, and a warm bath, were ordered."

Subsequently, it was deemed necessary that leeches should be applied to the temples, in consequence of an aggravation of the head-symptoms supervening. At this period the feet were cold, while the head was every now and then hot—a most unpleasant and unfavourable symptom. The tongue was of a dark reddish-brown hue. Dr. S. ordered that a table-spoonful of the following mixture be given every two hours.

℞. Infusi Cinchonæ et rad. Angelicæ ℥iv.
Mucilagin. Salep. ℥ij.
Acid. Muriatic. ʒj. M.

In the course of a day or two afterwards, symptoms of an inflammatory affection of the Larynx supervened. In some cases, this proves rapidly fatal perhaps by inducing *œdema glottidis;* while, in others, it terminates in *phthisis laryngea*—a disease which, as a matter of course, invariably causes death. The laryngeal affection is sometimes accompanied with most distressing spasmodic contractions of the muscles of the neck; and this spasmy state may extend to the pharynx, and give rise to symptoms of Hydrophobic dysphagia. Leeches, sinapisms, and mercurial frictions were ordered. These means had the effect of decidedly mitigating the local distress; and, for a day or two, the general symptoms appeared to be somewhat more favourable. But the amendment was only temporary. Castor and Musk were given repeatedly; and a bath, prepared with aromatic herbs, was ordered.

The patient died about the end of the third week of the fever. On *dissection*, were found "the usual appearances on the mucous coat of the intestines, considerable points of ulceration not yet cicatrised." The surface of the throat, pharynx, and the whole length of the œsophagus was covered with a lining such as we find in *angina gangrenosa*, or on the surface of ulcers affected with *hospital gangrene.*

In the Appendix, at the end of the volume, are some useful practical remarks in illustration of the various cases whose reports are given, or of the general history of Typhus. We have selected those which appear to be the most valuable.

*Diarrhœa and Cæcal pain not always present in Typhus.*

Although a certain degree of Diarrhœa is very generally present in this fever, it is by no means an invariable symptom. It may be absent, just as we sometimes see that there is no cutaneous exanthem in Scarlatina; no cough in Pneumonia, or in Phthisis, even when large vomicæ exist in the lungs; no vomiting in Scirrhus of the Stomach, &c. Occasionally, too, there is no tenderness whatsoever in the cœcal region. The cause of this may be, that the affected intestine is more deeply seated than usual in the pelvis, and may thus be withdrawn in a great measure from the pressure of the finger; or that the patient is more or less comatose, and does not understand, if he hears, the questions that are put to him. To return for a moment to the state of the alvine evacuations, we may remark that, if the fever proves fatal before the scabs (of the intestinal ulcers) are detached, there may be complete constipation present; and the tendency to this occurring will be increased, if there be any unusual tumefaction, as sometimes happens, of the ilio-cœcal valve. "You thus see (Dr. Schoenlein is addressing his clinical pupils) that, as I have frequently remarked, there is no individual pathognomonic symptom of the disease, and that no single phenomenon can determine anything with certainty. It is only the correlation of different symptoms one with another, and the mutual influence and reciprocal restraint and modification which they exert upon each other, that can lead to any satisfactory conclusions as to the therapeutic management, as well as the final issue, of each case."

The presence of a slight Diarrhœa in Fever is not to be regarded as necessarily an unfavourable symptom; it is such only, when it exceeds certain limits; and then it usually depends upon an irritation being established around the new formation (on the intestinal mucous membrane), which must become loosened and detached. When this is the case, the stools are usually watery and slimy. It will often be prudent to examine the intestinal evacuations with the Microscope; as in this way we shall be enabled to detect the presence of certain matters, which might otherwise escape detection. The admixture of pus-globules, and of epithelial scales, satisfactorily proves that the process of intestinal cicatrisation is not yet completed.

*Influence of Cutaneous Eruptions on the Development of Typhus.*

It has been asserted by some writers that the existence of Scabies is an almost sure preservative against the invasion of this fever. We have observed, says our author, that the presence of Impetiginous eruptions (especially those of a moist and purulent character) seems to afford considerable security against the *petechial*, but little against the *abdominal*, form of Typhus: our extensive experience in the years 1814 and 1815 led us to adopt this conclusion. The cutaneous eruption, it may be remarked, often exhibits very considerable modifications in its appearance, in those persons who are exposed to the febrile miasm. If, however, the fever is caught, then the eruption becomes more or less completely dried up; and this change may be either permanent, or only temporary during the existence of the febrile process in the system—the eruption again making its appearance, when the fever has passed away.

*Critical Evacuations—Urine—Perspiration.*

Much stress is repeatedly laid upon the observance of the proper critical days, more especially the 14th and 21st; so that the treatment employed may tend to promote, and not to check or arrest, any critical evacuation which Nature may design to take place at those epochs of the fever. It is very often observed that the Urine undergoes a very remarkable change about the 21st day of the disease; passing then from an *acid* to an *alkaline* condition, depositing the earthy phosphates, and occasionally also the carbonate of Ammonia. Whenever this takes place, we may regard it as a favourable symptom, and indicating the resolution of the fever. Should there be, at the same time, any tendency to free perspiration, it will be well to promote this effort of Nature by tepid or warm baths, the use of which will serve to promote the separation of the detached epidermis.

It occasionally happens that, on the day preceding the critical resolution, there is a marked exacerbation of febrile re-action: however, no artificial means should be used to accelerate this event. It is a natural process; and, the less it is interfered with, the better.

There is sometimes a very marked remission of all the febrile symptoms in Typhus fever upon the *seventh* day of the disease; the skin becoming moist, the urine turbid, the pulse quiet, and the tongue tolerably clean: perhaps also an Epistaxis takes place at the same time, and this might very reasonably be regarded as a *critical* hæmorrhage. On the morning of the eighth day, the patient may feel himself so well that he begins to think that he is convalescent; and yet, before evening, a new storm may arise, and from this date the nervous *stadium* or stage is often observed to commence. * * * * * *

Much mischief is often done by interfering too much with the course of the Typhous process in its stage of development, during the first week. The remedies, usually employed for this purpose, are Emetics and Purgatives; on the injurious effects of which we have already more than once spoken. They are more especially to be deprecated about the fourth day of the disease, when the intestinal eruption usually takes place; as they must then necessarily aggravate the inward irritation, just as stimulant applications to the skin would do in any Cutaneous Exanthem. Not only in genuine Typhus, but even in other diseases which happen to occur during the prevalence of this fever, we cannot be too cautious in the administration of emetics and purgatives; as there is then always a strong tendency in these diseases to assume a Typhoid character. Dr. Stokes has remarked that the injudicious use of purgative medicines, by patients recovering from Typhus fever, is apt in some cases to induce a perforation of the bowels, at the seat of the ulcers upon their mucous surface.

*Necessity of Caution in the Convalescent Stage—Causes of the great Emaciation after Fever.*

Too much attention cannot be paid to the state of the bowels, and of the urinary secretion, during the period of convalescence from fever—a period that may be dated from the cessation of the critical stage, which usually lasts from four to seven days. The intestinal ulcerations necessarily require some time before they are completely cicatrised; and the slightest irritation, whether from irregularity of diet, exposure to cold, and so forth, is apt to be followed by an immediate recrudescence of the pyrexial symptoms. Again, as long as there is the slightest tendency to the return of any evening feverishness, the state of the patient must be very narrowly watched. With respect to the urine, it is of importance that it be neither deep-coloured and having a strong acid re-action; nor inclined to be turbid, with an alkaline character; nor yet too pale and abundant, as it is apt to be in anæmic hysterical complaints.

The causes of the excessive Emaciation (some writers have termed it "typhous marasmus"), that is usually induced by Fever, are various. For example—1. There is the *lesion* of the *mucous membrane* of the intestines, and the consequent disturbance to the processes of Chylification and Absorption: the *villi* on the regenerated membrane are, for a length of time, much less numerous than before the ulceration. 2. Then again, the *mesenteric glands* are often more or less enlarged, if not inflamed; and the necessary consequence of which must be a partial obstruction to the free passage of the chyle through them. 3. Lastly, there may be a change or lesion of the nerves of Nutrition (of the solar plexus, more especially); and consequently a disturbance of the innervation of the organs which serve for this important function. (It seems strange that, in the enumeration of the causes of the excessive loss of flesh and strength usually experienced after an attack of Typhus fever, Dr. Schoenlein has here made no reference to the very serious alteration that the circulating fluids have undergone, in the course of the disease).

*Dissolved State of the Blood—Hypostatic Congestion of the Lungs.*

There are not many allusions in these Reports to the altered state of the circulating fluids in Typhus fever. Among the notes, we find the following short paragraph.

"In two cases, we found an unusual degeneration of the Blood: in the heart, there was not a trace of any fibrinous coagulum; the blood was still entirely fluid, although 24 hours had elapsed from the period of death: a large quantity had transuded through the walls of the vessels into the abdominal and thoracic cavities. This is the highest degree of what has been called the 'typhous dyscrasis of the blood:' in the present case, it seemed to be connected with the excessive evacuations that had been practised in the early stage of the fever."

One of the consequences of this morbid Fluidity of the Blood is a tendency to its accumulating and becoming congested in the posterior or decumbent parts of the body, and especially in the posterior portion of the pulmonary parenchyma—giving rise to what has been called a *hypostatic congestion of the lungs*. If, under such circumstances, the physician trusted entirely to the character of the auscultatory signs, he might be led into a most dangerous therapeutic error. A well-marked crepitating noise may be heard, as in genuine Pneumonia; and yet the use of any depletory remedies might prove rapidly fatal. Wine, bark, and ammonia must, as Dr. Stokes has justly remarked, be freely administered: whatever most effectually counteracts the dyscrasis of the blood, will be the best corrigent of the sanguineous infiltration of the lungs. The physician cannot be too much on his guard, against regarding the hyperæmic condition of certain organs in Typhus fever as examples of pure inflammatory action, and treating it accordingly by active depletory measures. The lesions of the intestinal mucous surface have been considered by some as of an inflammatory nature; and with what results is generally known. In reference to the thoracic symptoms also, the greatest caution must be employed; as, even in some cases where blood has been expectorated, blood-letting must on no account be resorted to, and by far the best remedies are bark and sulphuric acid.

*Tendency to Meningitis, and Suppuration of the Ear or Parotid Gland.*

The persistence of cephalic uneasiness, after the other phenomena of Typhus fever have disappeared—especially if, at the same time, there be great tenderness of one part of the skull upon pressure—has been generally regarded as a very unfavourable symptom. If the seat of the pain be in, or very near to, the ear, there is the danger of an internal suppuration taking place; not a few patients have died from this cause, after an attack of typhus.

In other cases, one or both Parotid glands become the seat of a troublesome suppuration. The tendency to these glands being affected varies very much in different epidemics. In some seasons we rarely meet with a case of this complication; in others, every typhous patient is more or less affected with it. The same remark holds true of Scarlatina: in one epidemic, Angina parotidea is an almost invariable symptom; in another, it is very rarely present. Even in Syphilis and Gonorrhœa we observe a similar diversity in their complications. Sometimes a bubo occurs in a large majority of cases; while, at other times, it is only of occasional occurrence.

An unusual or *excessive acuteness of the sense of Hearing* has been considered by many writers as a bad omen: P. Frank looked upon it as a fatal one. Without going so far as this, it is certainly a symptom that indicates, if not the actual existence of, at least the tendency to, some serious lesion of the encephalon or its membranes.

*Enlargement and Softening of the Spleen.*

It has been asserted by some writers that an enlarged and softened condition of the Spleen is a very constant, if not an essential, accompaniment of Typhus fever; and long lists of cases have been brought forward to support this pathological doctrine. The facts may be quite true; but the inference drawn from them appears to be erroneous. The occurrence of this complication depends very materially upon the locality or region where the fever exists. For example, it is very frequent in Holland and all marshy countries, where the malaria of agues abounds. Moreover, it is well known that, in most persons who have suffered severely from intermittent fever, the spleen is usually left more or less enlarged above its natural dimensions. We cannot wonder then that, if such persons catch Typhus—a disease in which there is always a tendency to visceral congestion—this enlargement, or hypertrophe as it has been called, should be considerably increased. But that it is by no means a constant accompaniment of this fever, we can have no hesitation in asserting: indeed, it is only in a few cases, according to our experience, that it is really present.

Andral has come to a similar conclusion on this question; viz. that the enlargement of the spleen is not a constant symptom, but only an accidental one—attributable in part to some peculiarity in a patient's constitution, and in part to local or epidemic influences. The complication, when it does exist, must be regarded unfavourably; for there is not unfrequently in those patients, in whom it exists, a great tendency to internal hæmorrhage, more especially from the stomach and intestines. Occasionally the parenchyma of the spleen has become so soft and lacerable that it has actually given way, and thus a large quantity of blood has been extravasated into the cavity of the abdomen. As a matter of course, this is invariably a fatal occurrence.

*Treatment of excessive Tympanitic Distension.*

The accumulation of gas within the bowels has occasionally been known to be so great as to lead to the laceration or perforation of their walls at one point. The remedy, which I have found to be most effectual for the relief of this symptom, is a clyster of cold water: the cold contracts the bowels, and diminishes the expansion of the gas; while the water absorbs a portion of it at the same time. The injection may be frequently repeated.

*Miliary Eruptions in Typhus.*

There are some epidemics, in which a cutaneous eruption or exanthem occurs in every case, so that it has even been regarded as characteristic of the Fever. It is generally associated with other critical phenomena in the Skin or in the Urine; and may readily be distinguished from the Rheumatic Miliaria, inasmuch as the fluid in the vesicles is in the former case alkaline—analogous to the alkalescence of the urine—whereas, in the latter, it is always more or less distinctly acid.

The miliary eruption in a Typhus fever is to be viewed as rather a favourable symptom than otherwise.

There is another form of miliary eruption which, upon a cursory examination, might be considered as of the same nature; but it is not. It consists of vesicles under the epidermis, usually very small, and almost microscopic; they are felt as minute papules or elevations through the skin. They contain not a liquid, but a gaseous (gasförmigen) fluid. Its true nature has not been ascertained; but it seems probable that it is Cyanogen; at least, in a somewhat similar phenomenon in the course of Smallpox, traces of hydrocyanic acid have been discovered.

This development of gas is usually accompanied with that of other unfavourable symptoms, indicative of the putrescence and dissolution of the blood.

The two sorts of Miliary eruption, alluded to above, should be very carefully distinguished, as their prognostic intimation is of very different import.

*Excessive Irritability during the Convalescent Stage.*

Not only after Typhus—especially that form of it in which the Sensorium is more particularly affected—but also after certain physiological acts or processes, such as Menstruation and Pregnancy, I have often had occasion to observe so great a degree of irritability, that, in not a few cases, most unpleasant consequences have ensued. In Italy, and other southern climates, it is often unsafe to bring any strong-smelling flower into the chamber of a puerperal woman, in consequence of the impression thereby made on the nervous system, and the high vascular re-action that is apt to follow: a state approaching to genuine *apoplexia nervosa* has been thus induced. Any slight impression on the mind or feelings is apt to do the same, when the system is in a highly-excitable mood. A sudden fear or surprise has often brought on an alarming Syncope, in a patient recovering from a severe attack of Fever. The disturbance of the sensorial functions after Typhus is sometimes so great, that the patient has completely forgotten all that he knew before the attack: it has even been known, under such circumstances, that an adult has been obliged to be again taught to read and write his own language.

*Is there really and truly a "Cerebral" Form of Typhus?*

If it be meant to be asserted that there is a distinct and well-defined form of this fever, which always sets in with the same phenomena (indicative of cerebral disturbance), and which might be appealed to as a *silhouette* or portrait that was applicable to each case, we can have no hesitation in contradicting the assertion. "Cerebral Typhus" is nothing but a modification of "Abdominal Typhus," where the nervous symptoms —those, namely, which point out an affection of the encephalon—are unusually dominant, and exert a certain modifying influence upon the general character of the typhoid phenomena; just in the same manner as we find in other cases that the bronchial symptoms are more prominent than usual; and then the fever might be called "Broncho-" or "Pneumo-typhus." But it would be a grievous error to suppose that, under any of these circumstances, the mucous membrane of the intestines is wholly unaffected and intact. The degree and extent of this lesion may be different in different cases; but it is present in all.

II. The paramount importance of the subject of *Fever* must plead our excuse for having devoted so large a space to this subject. We now proceed to examine the most interesting cases of *Pneumonia* and *Pleuritis*, recorded in these Reports. The first that we shall select is headed thus:

*Pneumonia in the upper Lobe of the right Lung—Simultaneous Affection of the Brain and Bowels—Difficulty of the Diagnosis—Absence of the subjective Phenomena—Superiority of the new Methods of Investigation—Inflammationes Occultæ—Recrudescence of the Pneumonia—Carditis—Recovery.*

A middle-aged man was brought into the hospital delirious and utterly incapable of giving any account of his ailments. His *head* was hot, his face flushed, and his eyes were somewhat bloodshot. The breathing seemed to be tolerably easy, and there was but little cough; in short, there was scarcely any subjective symptom that indicated the presence of any pneumonic distress. The left side of the *chest* sounded well on percussion and auscultation; but, on the right side, percussion elicited a dull sound over the upper part; and in this region there was a *respiratio tubaria*, and a dry crepitating noise perceptible. The *abdomen* was considerably inflated but soft; the cœcal region was somewhat tender, and gave out a rumbling noise on pressure. The pulse was rapid, small, and soft; and the skin was hot. Nothing was known as to the state of the urine and of the bowels.

From these facts, it was inferred that two organs were chiefly affected; 1, the upper lobe of the right lung, where there was inflammation in the stage of Hepatisation; and 2, the lower part of the intestinal mucous membrane. It was therefore suspected that the Pneumonia was associated with a typhous condition. The cerebral phenomena were viewed as merely consecutive and incidental: they might be the result of the abuse of spirituous drink, as the patient's breath smelled somewhat of it. Accordingly, some blood was taken by cupping over the right lung, and a venæsection was ordered to be practised in the evening, if the inflammatory symptoms became more decided. The following mixture was also prescribed:

℞. Infusi Digitalis (gr. x.) ℥iv.
Mucilag. Salep. ℥ij.
Natri nitrici ʒij.
Syrupi simpl. ℥j. M.

During the night, the patient was very restless, and could scarcely be kept in bed. Six ounces of blood were therefore taken, early next morning, from the arm. It was now ascertained that the patient had been ill eight or nine days, and had taken some Epsom salts, which had acted pretty strongly upon the bowels: the intestinal affection might therefore be regarded rather as a medicamental result, than an original symptom of the disease. The concentration point, the key of the riddle, doubtless lay in the organs of Respiration; and here we may make the general remark, that the difficulty of Diagnosis consists not so much in the mere discovery of the symptoms of a disease, as in the due appreciation of their relative importance and mutual bearings.

The cephalic symptoms were decidedly relieved by the remedies; and then the *subjective* symptoms of the thoracic disorder immediately became, as usual, much more distinct. This is a common occurrence, and deserves to be attentively considered. Schoenlein mentions briefly another case, in which the patient appeared to be quite free from any affection of the organs of breathing as long as he was in a delirious state; but no sooner did this subside, than the symptoms of an active Pneumonia at once became strongly marked.

It was necessary to repeat the venæsection to a small amount: the same internal remedies were continued. Every thing promised a favourable issue; as the state of all the great cavities of the body seemed now to be much more easy and comfortable. The diarrhœa and irritation of the bowels had so entirely ceased, that an enema was required occasionally. The sound, on percussing the right side of the chest, was still dull; but the crepitation was replaced anteriorly by a mucous rale; a change that indicates a resolution of the inflammatory action. The former of these sounds however, accompanied with bronchial respiration, was still heard in the axillæ, and also behind, over the scapulæ: at these parts, therefore, the pulmonary parenchyma was considered to be still in a state of hepatisation. The pulse had become much more favourable, and the skin and kidneys had evidently begun to throw off critical evacuations.

When every thing was going on favourably, a very unexpected recrudescence of the pneumonic symptoms took place, in consequence of the patient having left his bed while he was freely perspiring. Blood was immediately taken, both from the arm, and also by cupping over the seat of the pain; the use of the *Digitalis* and *Nitre* was resumed, and half a grain of *Calomel* and of the *Sulphur. aurat.* was given morning and evening.

Such relapses must always be regarded on the whole with an unfavourable eye; especially when, as in the present case, there is reason to apprehend the supervention of any Cardiac affection—indicated by a rapid, irregular, and somewhat intermittent pulse, and the presence of a "follicular rushing" sound over the origin of the Aorta, at the left edge of the sternum. A large blister was therefore applied at this part. On the next day, the patient was decidedly better, and the following mixture ordered to be taken, in spoonful doses, very frequently:

℞. Decocti Salep ℥vj.

Nitri ʒss.

Ammon. Muriat. ʒj.

Solution. liquirit. ℥ss. M.

The remarks made, on the occasion, by Dr. Schoenlein are to this effect:—

"I will here attach no great importance to, or lay much stress upon, the *subjective* phenomena; as, for example, on the circumstance that the patient has slept well, coughed little, and feels himself better. Such symptoms are only valuable when they accord and coincide with an amendment in the state of the *objective* signs. That such is the case in the present instance, is proved by the circumstance that it is now only over the posterior part of the upper lobe of the right lung that there is any trace of moist rattle to be heard, and that the cardiac abnormal sound is no longer perceptible; the pulse having at the same time become less rapid, although it is still somewhat irregular.

"It became a question whether the irregularity of the pulse might not be owing, in part at least, to the action of the Digitalis; but then this action could never have occasioned the objective cardiac phenomena; moreover the frequency of the pulse was accelerated rather than otherwise; nor were there any of the other symptoms that are apt to be induced by the use of this drug."

The patient eventually recovered.

Upon the important subject of the *Co-existence of Carditis with Pneumonia,* we find the following observations appended in a note.

"You have seen several cases, where, on the relapse of a pneumonic attack, inflammation of the Heart was found to be co-existent. I believe that such a complication is not of unfrequent occurrence; and I need scarcely add, that it adds very seriously to the danger of the malady. Whether the development of cardiac inflammation under such circumstances is at all facilitated or promoted by the previous administration of Digitalis—a drug which has so lowering an effect on the action of the heart—is a question that deserves some consideration, and which we are not quite prepared to answer one way or the other. It may not be altogether undeserving of notice that, in Chlorotic girls, whenever inflammatory action is set up, there is a marked tendency to the Pericardium and Endocardium becoming affected. If such be the case, the operation of very potent antiphlogistic measures may induce the same predisposition. But, without seeking to find out the cause or reason why such things are so, it may be sufficient for the practitioner to know that a Heart-affection is very frequently associated with a relapse of Pneumonia."

### *Value of Auscultation and Percussion.*

The opponents of the new method of investigation allege—and its most zealous advocates do not deny the charge—that it is still very imperfect; but surely such imperfection forms no sufficient reason for its condemnation or rejection. For example, it is quite true that, when inflammation exists in a central portion of any lobe of the Lungs, and when the affected part is surrounded with healthy parenchymatous structure, the auscultatory signs are of comparatively little value. But, even under these circumstances, our diagnosis as to the Seat of the morbid process may be very materially assisted by the very absence of the pathognomonic acoustic phenomena. Thus, if there be present the *sputa cruenta* with the other subjective symptoms of Pneumonia, and yet auscultation fails in detecting any abnormal sounds, we may reasonably conclude that the inflammation must be seated in the central and deep-seated part of the affected lobe or lobes. Let it ever be remembered that no judicious physician will ever trust exclusively to the auscultatory signs, in this, or in any other disease; the functional symptoms and the state of the bronchial secretion will always be attentively examined. But, because the former may occasionally be apt to mislead, surely this is no sufficient argument for rejecting their assistance altogether.

How important is the use of Auscultation in assisting our diagnosis, where the patient may be delirious, or otherwise incapable of giving any account of his feelings! We are, every now and then, meeting with cases where the *subjective* symptoms of Pneumonia may be almost entirely absent, (the breathing being apparently quite free, and no cough existing, nor any pain* in the chest complained of), and yet in which there cannot be a doubt, from the nature of the auscultatory signs, that inflammation of the Lungs is present in the lungs. Dr. Stokes very judiciously recommends that the state of the thoracic viscera should never remain unexplored in patients labouring under *delirium tremens* or Typhus fever, whether there be any obvious symptoms of inflammation present or not.

There is another condition in which the use of auscultation may prove of the greatest benefit in directing the medical treatment; and that is when all the active subjective symptoms have subsided, and the patient has turned, as it were, the corner in his advance on the road to health. Let it never be forgotten that, as long as there is any mucous rale perceptible, we have good reason to believe that the inflammatory process has not entirely ceased, and we should still carefully avoid the exhibition of stimulants and tonics. Much harm is frequently done by having recourse pre-

---

* The degree of *pain* in Pneumonia depends a good deal upon the part of the lung that is affected, according as it moves little or much up and down, in the acts of breathing. Hence it is, that it is usually more severe when the middle, than when the upper or lower, lobe is inflamed.

maturely to the use of these means after an attack of Pneumonia. A far safer practice, on the whole, is to continue the use of such medicines as *nitre, sal ammoniac,* and *sulphur. aurat.* until all the dregs of the disease are entirely got rid of.

The *seat* of the *pain* in Pneumonia cannot be trusted to, by itself, as a sure guide as to the *seat* of the *inflammation.* Stoll and other writers record many cases in which dissection proved that the inflammation was on the opposite side from that on which the pain was felt during life. It is only by the use of the Stethoscope that we can attain to any exactitude in our diagnosis as to the *seat* of a Pneumonic attack.

In a former part of this article, we have alluded to the modifying influence of an Agueish principle on the course of Typhus fever. A somewhat similar change is observed to result from the operation of this principle on the symptoms and general character of several thoracic diseases. In one of the cases of Pneumonia recorded in these Reports, the patient had been long subject to Ague. Dr. Schoenlein availed himself of the opportunity, thus presented to him, of pointing out the peculiar idiosyncrasy of constitution which this circumstance is apt to engender:—but first let us notice his remarks on a question that has been much occupying the attention of some French physicians of late, the *Antagonism between Intermittent Fevers and Phthisis.*

"Although it would seem that these diseases exclude each other (at least, in a very great measure) so that the latter is seldom met with in those places where the former prevails, it is unquestionably true that persons—who, after having long suffered from Ague, have left the malarious district and have gone, seemingly quite cured, into another region—are exceedingly liable to being attacked with catarrhs; and these catarrhs not unfrequently terminate in confirmed, and often galloping, consumption, although there be no hereditary or constitutional predisposition to the disease. I first observed this fact in those Swiss, who had returned from the ague-districts of Holland. In cases of this sort, according to my experience, the tuberculous disease is generally most developed in the *left* lung—corresponding therefore with the side on which the Spleen is situated—and most frequently too, in it's lower lobe. There was always more or less splenic enlargement present at the same time, in the patients affected in this way. A striking illustration of the antagonism, that exists between Intermittent fever and Phthisis, will be found in the medical geography of Gasterland (situated between the lakes of Zurich and Wallenstadt). This district used to be the constant seat of agues, in consequence of the land being frequently overflowed; subsequently it has become quite dry. The Agues have left it, but their place is taken by Phthisis, a disease which was formerly almost quite unknown."

In reference to the case of Pneumonia, to which allusion was made above, the following observations are introduced by our author.

"In such persons, various diseases, with which they may happen to be affected, are usually more or less decidedly modified, so that their course in reference to symptoms and crises is considerably altered from that which we generally observe them to pursue. This is a point which is of the highest importance in the practice of medicine, although it has not met with the attention which it deserves. A somewhat analogous change is observed in respect of Plants; which, it is well known, undergo such considerable modifications from variety of soil, difference of exposure to the sun and so forth, that mere *varieties* are not unfrequently apt to be mistaken for different *species.* In the same manner, the characters of diseases may be very materially modified, according to the nature of the patient's constitution in which they become developed. How difficult, for example, it is to eradicate a syphilitic taint from a scrofulous habit of body! and who does not know the extreme tendency of gonorrhœa, and other mucous discharges, to become chronic in strumous persons. In Ophthalmia too, how much does the obstinacy and intractableness of the symptoms depend upon the idiosyncrasy, natural or acquired, of the invalid! Now, if this remark be true of the eye, there is every reason to believe that it is equally applicable to every other organ of the body, although in none can the progress of the disease be so well observed as in it. We might allude also to the difference in the nature of morbid formations and secretions observable in different constitutions. The coagulable lymph, that is effused during an attack of Pleuritis or Peritonitis in a scrofulous patient, becomes very often the seat of tuberculous matter deposited in its interstices."

But to return to the complication which suggested these remarks—viz. that of an Agueish tendency with an Inflammatory disease,—let us briefly notice some circumstances which may suggest to the mind of the physician the suspicion of such being the case. During the full activity of the phlogistic symptoms, nothing unusual may be observed; it is when these have begun to subside, that the peculiarity is usually discernible. Perhaps everything may be promising that the severity of the inflammatory attack is past, and that the disease is fairly subdued, when the patient becomes hot and feverish (with, or without a previous chill) in the afternoon, complains of headache, and may even be delirious, the pulse being much accelerated all the while. These symptoms continue more or less severely until about midnight, when they subside; the patient then probably falling asleep, and awaking in the morning much better. Now such a febrile invasion and remission may be repeated day after day, for some time; and, unless the physician forms a correct diagnosis as to the real nature of the case, he may unfortunately persevere in the use of those very means which will inevitably render the Intermittent type more and more difficult of removal. The sooner, as a matter of course, that Bark is administered in such cases, the better. There may, indeed, still remain the dregs, so to speak, of the inflammation; and therefore the administration of this remedy may require to be accompanied with the cotemporaneous use of appropriate antiphlogistic and derivative means, as cupping, blisters, and so forth.

P. Frank has some useful remarks on this subject in his *Interpretationibus Clinicis*. This writer remarks that, whenever the paroxysm of an Intermittent fever is in the habit of daily coming on sometime after noon, we have reason to suspect that it is not a genuine and simple Quotidian that we have to deal with, but that there is some other morbid action coexisting at the same time. In cases of complicated Intermittent, the paroxysm also very frequently takes place without any cold stage; and the occurrence of any distinct crises by the skin or kidneys, under such circumstances, is always more rare and imperfect than in simple Agues. Thus the Urine may remain of a deep colour, without any change taking place, when the febrile paroxysm is not present; and if a critical evacuation does really occur, this will perhaps be the genuine crisis of Inflammation, as a hæmorrhage from the nose or some other part of the body.

Before passing on to the subject of Pleuritis, it may be as well to find a place here for the following remarks on two practical points, viz. 1, the employment of *Tartar Emetic in Pneumonia;* and 2, the treatment of a peculiar *status nervosus*, which is apt to occur in some cases of this disease.

With respect to the effects of the Tartrate of Antimony in thoracic inflammation, M. Schoenlein is by no means inclined to go nearly so far in its praise as some recent writers, who pretend that it may almost supersede the use of blood-letting. That it is indeed a most powerful auxiliary, no experienced physician will deny; but it should rarely be trusted to alone. When a Pneumonia has already lasted for some days, and still continues in spite of active depletion, the exhibition of the tartar emetic cannot be too highly praised; but then only after Venæsection has been duly practised. Again, whenever the biliary organs are much deranged at the same time, great benefit may reasonably be expected from the judicious employment of this potent remedy. The contra-indications to its use are the existence of any symptoms of an inflammatory or irritated condition of the gastro-intestinal membrane (inflammation of the cardiac orifice of the stomach is a not unfrequent accompaniment of Pneumonia), or any tendency to Diarrhœa. In old people more especially, it requires to be used with caution, as in them it is apt to induce a Marasmus of the digestive organs. M. Schoenlein is not friendly to the exhibition of the very large doses recommended by some writters. His usual formula is a solution of from two to six grains in as many ounces of water; of this mixture, one-half is to be taken at once, and then a table-spoonful of the remainder every half-hour or so.

The *nervous condition*, which not unfrequently comes on in cases of severe Pneumonia, in consequence of some disturbance of the circulation,—and which is indicated by a frequent, small, and oppressed (unterdruckten) pulse, a blueishness of the countenance, coldness of the extremities, delirium, &c.—should always be cafefully distinguished from that state wherein the attendant fever is of a torpid or typhoid character from the very beginning, as is the case in genuine *Pneumonia typhosa*. Unfortunately both of these conditions have been at different times described under the general appellation of *nervous*. The physician must be on his guard that he is not deceived by the symptoms in the first of these cases, and thereby be led prematurely to suspend the use of antiphlogistic, and have recourse to that of stimulant, remedies. We should steadily persevere in the employment of the former, although certainly it may be wise to modify them according to individual symptoms.

One of the best remedies under such circumstances is *Digitalis*, in the form of infusion, to be given in frequently-repeated doses: some of the *Aqua lauro-cerasi* may often be added to it with advantage. It lowers the action of the heart, and at the same time serves to stimulate the urinary secretion. Small doses of *Calomel* and *Sulphur. aurat.* night and morning, may also be of great service, at the same time.

III. The class of cases, which next attracts our notice, is that of Pleuritis, and—its not unfrequent consequence—the effusion of a serous or sero-purulent fluid into the cavity of the chest. In the remarks which immediately follow, the attention of the reader is drawn to a peculiar symptom which may perplex his diagnosis in some instances.

*Complication of Spinitis with Pleurisy.*

We occasionally meet with cases of Pleuritis, which are accompanied with severe pain in the back, and stiffness of the vertebral column: these phenomena *may* depend upon an extension of the inflammation to the investing sheath of the spinal-marrow. Allan has described this form of the disease, in his *Synopsis Medicinæ Practicæ*, under the name of *Pleuritis Postica.* A case of this sort occurred in Schoenlein's clinical practice a few years ago. A labouring man was admitted with all the symptoms of Pleuro-pneumonia on the left side: there was reason to believe that the inflammation extended to the diaphragmatic pleura. At the same time, the patient complained of a severe dragging pain in the sacral region, much increased on pressure and motion, along with a feeling of tension around the abdomen, and a sense of formication along the left lower extremity. The patient was freely bled from the arm, and also by means of cupping over the loins, and ultimately recovered. In reference to that case, Dr. Schoenlein made the following remarks at the time.

"These severe pains in the spine are not unfrequently mistaken and treated as if they were of a Rheumatic origin; and perhaps, before their real nature is discovered, paralysis has already set in. Exposure to cold and wet is the most frequent cause of this dangerous lesion. I had repeatedly occasion to witness cases of this complication at Wurtzburg among the soldiers who kept sentry upon the walls at night, exposed to the cold sharp winds: in some instances, the attack assumed a tetanic character from the commencement. I have also met with cases among persons who were travelling on foot through Switzerland, and who perhaps had remained all night upon the summit of Mont Rigi, in order to witness the rising of the sun in the morning. One of these cases proved fatal.

"We need not say how very important it is to distinguish any case of Spinitis from one of mere Rheumatism. The pain will be found to be seated not in the muscles, but fixed at some part of the spinal column; and it is always much aggravated by pressure and motion. A feeling of constriction in the abdomen, as if a band was drawn tightly around it, is a symptom that is generally present; and likewise that of numbness or perhaps of formication in one or both of the lower extremities. The act of walking is always more or less painful and difficult; and there is very generally some disturbance in the excretion of the urine. If the cervical portion of the spine be the seat of the disease, then there is usually a sense of constriction in the chest; perhaps also a spasmodic cough, or, it may be, nausea and vomiting; in many instances, too, a greater or less degree of paralytic weakness of the upper extremities. As to the treatment of all such cases, we should have recourse to blood-letting (especially local), the inunction of mercurial ointment over the affected part, and the exhibition of large doses of calomel, to which some jalap should be added: subsequently, the use of warm-baths is of the greatest service."

CASE.—*Pleuritis mistaken for Rheumatism—Effusion into the right Sac of the Pleura—Indications for having recourse to Paracentesis—Stimulation of the Normal Secretions—Narcotism from Digitalis—Recrudescence of the Pleuritis—Recovery.*

A man, 26 years of age, had been seized, three weeks before his admission into the hospital, with shivering followed by heat, and with sharp pain on the right side of the chest, increased by deep inspiration, and by any movement of the arms. The case had been mistaken for one of Pleurodyny—a mistake, by-the-bye, that is not very unfrequent—and treated with tartrate of antimony, cupping and blisters ; remedies, however useful in themselves, not potent enough for the dangerous disease that was actually present. When admitted, the breathing of the patient was not much distressed, except when he attempted to lie on his left, or sound, side. The right side of the thorax appeared to the eye somewhat fuller and more arched out than the other. The whole of this (the right) side, with the exception of the space between the clavicle and second rib, was dull on percussion, and no distinct respiratory murmur was to be heard over it. There was a slight cough, which (pressure had the same effect) caused pain between the fifth and sixth ribs. The liver was to be felt lower down than usual: in some cases of effusion into the right pleura, this viscus has been known to be thrust down as low as the ilium.

The nature of the present case was obvious : a pleuritic attack had terminated, by a pseudo-crisis, in a serous effusion. As there was still a feverish frequency of the pulse, and the urine was also deep-coloured, it was deemed not unlikely that the inflammatory action had not entirely subsided. Accordingly, a small quantity of blood (which proved to have the buffy coat) was taken from the arm, and the cupping-instruments were applied over the spot where the pain was felt. At the same time, a mixture—composed of infus. Digitalis (℈j.) ℥iv., Mucilag. Salep. ℥j., Nitri ʒij. (the *liquor kali acetici* was subsequently added), and syrup ℥j., was ordered to be taken in frequently-repeated doses ; and the right side of the thorax was well rubbed with an ointment, consisting of *ung. hydrarg.* and *ung. pot. hydriod.*, to which some *oleum hyosciami* and *oleum juniperi* were added. As the medicine did not act sufficiently upon the bowels, the *tartarus boraxatus* (℥ss.) was substituted for the nitre.*

It is certainly very surprising how little the breathing is distressed in some cases of extensive pleuritic effusion. Instances have been known where the patient has scarcely suspected that any thing was amiss in his chest, until he happened to observe that his heart was beating on the wrong side, to the right of the sternum. Perhaps the only inconvenience that he had hitherto experienced, might be a slight oppression in his breathing upon going up stairs, or in the evening after a full dinner.

It is sometimes not very easy to determine which is the best emunctory —the bowels or the kidneys—by which the evacuation of the effused fluid should be sought to be promoted. Much must depend upon what seems to be the tendency of Nature in each individual case. Most frequently, certainly, the effort is made by the kidneys ; and here it deserves to be noticed, that the character of the urinary secretion is found to vary much in different instances—according, it would seem, to that of the effused fluid. When this is serous, the Urine is usually merely increased in quantity, and but little altered in its qualities ; while, in other cases, it is found to contain a quantity of mucus, or even of pus, when the pleural effusion is of a purulent character. In this manner, therefore, we may often be enabled to predict the nature of the fluid effused into the cavity

---

* Upon a previous occasion, Schoenlein remarks that he has often found the milder aperients, and especially the *Syrupus Rhamni*, answer a great deal better in dropsical cases than many of the drastic purgatives in ordinary use, such as Gamboge, Elaterium, &c.

of the thorax, by attending to the appearances exhibited by the urine. When a purulent fluid is present, there is usually more or less of evening hectic; and it is found that the discharge of such a fluid with the urine generally occurs during the night. The irritation of the urinary passages from the elimination of pus by the kidneys is sometimes very considerable, and may even induce slight Nephritis. In a few cases of pleural effusion, we have seen a decidedly purulent discharge from the bowels; but this is much less frequent than from the kidneys.

In reference to the use of Digitalis, our author remarks that its effects require to be very attentively watched, especially in old people; for in them it is apt to induce not only an alarming weakness, but a positive wasting and *marasmus*—probably by its acting injuriously upon the nervous system and the organs of digestion. The cautious physician, therefore, will never omit diligently to ascertain the state of these parts, during, and for some time after, the administration of this potent medicine. The preparation, which Dr. S. considers one of the best, is the *acetum digitalis;* the exclusion of which from the late edition of the Prussian Pharmacopœia, he very much regrets.

When all, or nearly all, the pleural effusion in the present case was absorbed, the patient began to complain of pain in the intercostal space between the eighth and ninth ribs on the right side. That the albuminous or fibrinous contents of the effusion—and these are often in the state of coagula or large flocculi—may act in some degree as foreign bodies, when all the fluid portion has been drawn off or absorbed, is not at all improbable; and perhaps such was the case in the present instance. However this may be, it will always be prudent to arrest at once the very earliest symptoms of a serous inflammation; for it is truly remarkable with what quickness (in the case of the Peritoneum still more so than in that of the Pleura) the disease spreads. Accordingly, a small quantity of blood was taken from the seat of the pain by cupping, and the use of the *Digitalis* and *Nitre* was resumed, until all the symptoms of uneasiness were removed. The patient eventually was restored to health.

In the next *case,* the Pleuritic Effusion (the presence of which had been quite overlooked by the medical man who first saw the patient) was on the left side, and to such an extent that the Heart was pushed considerably over to the right side, so that it was felt to beat behind and at the right edge of the sternum. The usual subjective and objective symptoms were tolerably well-marked. The diaphragm was considerably pushed down, so that the epigastric region was more prominent than in health, and pressure there occasioned a sense of oppression and distress.

Nearly the same remedies were used in this, as in the preceding, case. As there was reason to believe that there was still some degree of inflammatory congestion in the lower lobe of the left lung, a small quantity of blood was taken by cupping. The following mixture was prescribed:—

℞. Infus. herb. Digitalis (gr. xij.) ℥iv.
Nitri depurati,
Kali acetic. āā ʒij.
Syrupi Mannæ, ℥j. M.

A table-spoonful to be taken every two hours: Selzer water for common drink. The left side was ordered to be rubbed with an ointment composed of mercurial ointment, hydriodate of potash, and digitalis.

Two or three days subsequently, the bronchitic symptoms being aggravated, the patient was bled from the arm to a small extent and with decided relief. An attack, having somewhat of the character of *delirium tremens,* supervened, and necessarily rendered the prognosis rather more unfavourable. It became, therefore, very seriously the question whether we should continue to trust to the action of the remedies in use for the removal of the water, or have recourse at once to paracentesis for its evacuation. The results of Dr. Skoda's experience in this operation have been so very unpromising* (two-thirds of his patients died and generally too in consequence of a fresh accession of the Pleuritic disease), that there is certainly not much encouragement for its performance. Fortunately, it was deemed wise in our case to defer having recourse to it; for the thoracic symptoms began speedily to subside under the operation of the remedies employed. On the whole, Dr. Schoenlein seems to be anything but favourable to the operation of Paracentesis in cases of pleuritic effusion, more especially when there is reason to believe that flocculi of coagulable lymph are present, or floating about in the effused fluid; for, in his opinion, the presence of these flocculi not unfrequently excites inflammation of the pleura, after the serum has been drawn off by puncture. It may, indeed, be objected that the same thing must take place whether the fluid part of the effusion be removed by absorption or by paracentesis; but to this it may be answered that, in consequence of the slow and gradual removal of the fluid in the former case, the contact of the flocculi with the surface of the diseased pleura can never excite so much irritation as when the water has been suddenly drawn off all at once by operation. Moreover, it seems not at all improbable that they may undergo a partial dissolution and absorption, just as we observe to take place with the coagula of blood in the Brain in certain cases of Apoplexy.

The progress of this case was, on the whole, in spite of several unpleasant occurrences, pretty favourable. The quantity of the effusion gradually diminished under the use of the mixture (a drachm of the Muriate of Ammonia had subsequently been added to it), so that the heart began to resume its natural position on the left side. The patient left the hospital, before he was completely cured: the level of the effusion however had already sunk below the left nipple.

As illustrative of the general question of Pleuritic effusion, and of the propriety of not hastily having recourse to a surgical operation for its withdrawing, the following very interesting Report deserves a place here.

### *Phthisis—Rupture of a Vomica—Effusion of Gas and Air into the Pleura—Paracentesis Thoracis—long survival of the patient.*

"The displacement of the heart, *Ectopia Cordis,* from the effusion of fluid into the left sac of the pleura, was very conspicuous in a case of Hydro-pneumothorax—the result of a phthisical cavern in the upper lobe of the left lung becoming suddenly ruptured—which once occurred in Dr. Schoenlein's practice. The heart was found beating at the right side of the sternum; and, according to the patient's statement, this had taken place in the night, immediately after a

---

* Oester. Med. Jahrbucher. Januar—Juli, 1841.

severe fit of coughing. The place of the rupture was probably closed, for a time at least, by a false membrane. There was but little fever present, and the dyspnœa was so inconsiderable that it was deemed prudent to delay the performance of paracentesis for some time, and to try the effects of remedies in causing the absorption of the effused gaseous and purulent matters. Accordingly, the *infus. Digitalis* was prescribed, and the affected side was diligently rubbed with an ointment, composed of *ung. mercurial.* ʒij., *ung. kali hydrojod.* ʒj., and *ung. Digital.* ʒj. At first, the use of these means seemed to be attended with benefit; but this was only temporary. A decided fulness, amounting almost to a pointing, became perceptible in the ninth intercostal space on the left side. Here, therefore, an incision was made into the cavity of the pleura; between six and seven quarts of purulent matter flowed out. The immediate effects of the operation were satisfactory; for the dyspnœa and oppression were very much relieved. It seemed however that the rupture in the lung again opened shortly afterwards, as a fresh accumulation of pus took place in the pleural cavity: and the wound was therefore kept open, to permit the free exit of whatever was effused. As might be expected, the patient gradually became worse and worse. He survived however for six months after the bursting of the vomica, and for nearly two after the performance of the operation. On dissection, the left lung was found much shrivelled and contracted; on its under surface there was a rupture, so that a free communication was established between one of the bronchi and the cavity of the pleura, which still contained an admixture of gaseous and purulent matters: the right lung was full of tubercles."

Here we must close for the present our analysis of this truly valuable work: we shall probably resume it in our next Number, as there still remains more than a half of its contents unnoticed.

